# A Natural Flavone Tricin from Grains Can Alleviate Tumor Growth and Lung Metastasis in Colorectal Tumor Mice

**DOI:** 10.3390/molecules25163730

**Published:** 2020-08-15

**Authors:** Grace Gar-Lee Yue, Si Gao, Julia Kin-Ming Lee, Yuk-Yu Chan, Eric Chun-Wai Wong, Tao Zheng, Xiao-Xiao Li, Pang-Chui Shaw, Monique S. J. Simmonds, Clara Bik-San Lau

**Affiliations:** 1 Institute of Chinese Medicine, The Chinese University of Hong Kong, Shatin, New Territories, Hong Kong, China; graceyue@cuhk.edu.hk (G.G.-L.Y.); shirleygao@cuhk.edu.hk (S.G.); julialee@cuhk.edu.hk (J.K.-M.L.); cwwong_eric@cuhk.edu.hk (E.C.-W.W.); Zhengtao@cuhk.edu.hk (T.Z.); pcshaw@cuhk.edu.hk (P.-C.S.); 2 State Key Laboratory of Research on Bioactivities and Clinical Applications of Medicinal Plants, The Chinese University of Hong Kong, Shatin, New Territories, Hong Kong, China; 3 Li Dak Sum Yip Yio Chin R&D Centre for Chinese Medicine, The Chinese University of Hong Kong, Shatin, New Territories, Hong Kong, China; cyy1010yyc@gmail.com; 4 School of Life Sciences, The Chinese University of Hong Kong, Shatin, New Territories, Hong Kong, China; 5 Royal Botanic Gardens, Kew, Richmond, Surrey TW9 3AB, UK; m.simmonds@kew.org

**Keywords:** colorectal cancer, tricin, flavone, grains, anti-tumor, anti-metastasis

## Abstract

Tricin, a flavone isolated from rice bran, has been shown to be chemopreventive in a colorectal cancer (CRC) mouse model. This study aimed to illustrate the inhibitory activities of tricin in colon cancer cells and in a metastatic CRC mouse model. BALB/c mice injected with mouse Colon26-Luc cells into the rectum wall were treated with tricin (37.5 mg/kg) daily for 18 days. Orthotopic colon tumor growth and metastasis to lungs were assessed by in vivo bioluminescence imaging. Results showed that tricin suppressed Colon-Luc cells motility and downregulated phosphorylated Akt, Erk1/2 and NF-κB expressions of human colon cancer HT-29 cells. While tricin treatment suppressed tumor growth and lung metastasis as well as altered the populations of myeloid-derived suppressor cells and regulatory T cells in spleens. In summary, the tumor microenvironment modulatory and anti-metastatic effects of tricin in colon cancer mouse model were shown for the first time, suggesting the potential development of tricin-containing food supplements for CRC patients.

## 1. Introduction

Colorectal cancer (CRC) prevalence in different populations is increasing and it now shows the second highest rate of cancer mortality worldwide [1]. New prevention strategies are thus urgently needed to reduce the burden of CRC. CRC is influenced by genetics, lifestyle, diet, intestinal microbiota, etc. The awareness of maintaining colon health through diet and health supplements consumption draws great attention. Diet and lifestyle modifications for patients are highly recommended in order to alleviate the impact from colon disorders on the quality of life. A healthy pattern diet, generally characterized by high intake of fruits and vegetables, nuts and legumes, fish and other dairy products, was associated with lower CRC risk [2]. Besides, a meta-analysis revealed that one of the food items that has convincing epidemiological association with colorectal cancer risk reduction is whole grains [3].

**Figure 1:**
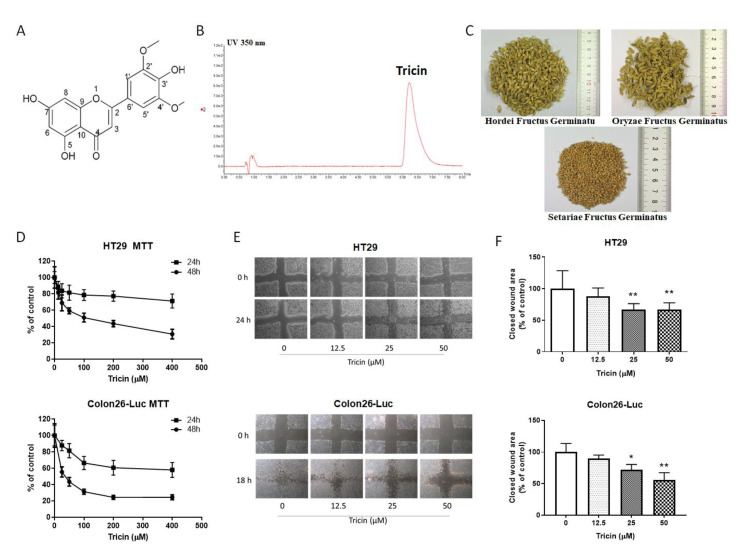
(**A**) Chemical structure of tricin. (**B**) UPLC chromatogram showing the peak of tricin detected at UV350 nm. (**C**) Photos of Hordei Fructus Germinatu, Oryzae Fructus Germinatus and Setariae Fructus Germinatus. (**D**) Effects of tricin on viability of HT29 and Colon26-Luc cells by MTT assay. Data were expressed as the mean percentage of the control (4 independent experiments with 4 replicates each, mean ± S.D.). (**E**,**F**) Effects of tricin on motility of (**E**) HT29 cells and (**F**) Colon26-Luc cells by scratch wound assay. Data were expressed as the mean percentage of untreated control (4 independent experiments with duplicate each, mean + S.D.). Statistical differences were determined by one-way ANOVA, followed by Tukey multiple comparisons test, with * *p* < 0.05, ** *p* < 0.01 against untreated control.

Grains (rice, corn, barley, wheat, oat, millet, etc.) are staple foods worldwide. Grains belong to the monocot Poaceae family and are widely cultivated to obtain the edible components of their fruits. Whole grains have an outer bran coat, a starchy endosperm, and a germ. Concerning the medicinal values of grains, previous studies have demonstrated that active ingredients such as polyphenols in sorghum bran [4], protein and peroxidase in millet bran [5], soluble phenolic content [6] and oil [7] of rice bran, and fermented rice bran-containing diet [8], could have suppressive effects on colitis and colon cancer cell growth. The bioactive phytochemicals present in rice bran such as ferulic acid, tricin, β-sitosterol, g-oryzanol, tocotrienols/tocopherols, phytic acid are regarded to possess chemo-preventive potential [9]. In fact, one of the flavones found in brown rice and rice bran, tricin (Figure 1A,B), was reported to inhibit growth of breast and colon cancer cells in our early study [10]. The subsequent animal studies demonstrated the interference of tricin on carcinogenesis of colorectal cancer in *Apc^Min^* mouse model [11,12] and the inhibitory effects of tricin on inflammation-related colon carcinogenesis mouse model [13].

Tricin was found to be the most abundant among the commonly reported flavonoids in bran [14]. Although wheat hull [15] and rice bran [16] are the rich sources of tricin, they are seldom consumed in regular diet. Nonetheless, rice bran such as Oryzae Fructus Germinatus can be used as Chinese medicines. In fact, there are several kinds of grains used as Chinese medicines which are often prescribed by Chinese medicine practitioners, namely Hordei Fructus Germinatus (germinated barley or malt), Oryzae Fructus Germinatus (germinated rice) and Setariae Fructus Germinatus (germinated millet) (Figure 1C). According to the Chinese Pharmacopoeia 2015, these three types of medicinal grains are for digestive problems, such as food retention, spleen deficiency with reduced food intake, and promote digestion [17]. A clustering and correlation analysis showed that Setariae Fructus Germinatus (SFG) was frequently used for advanced colorectal cancer patients, whereas Hordei Fructus Germinatus (HFG) and Oryzae Fructus Germinatus (OFG) were commonly used for improving cancer patients’ appetite [18]. Therefore, based on the traditional usage of these medicinal grains in gastrointestinal disorders and the presence of the chemopreventive flavone tricin in HFG (in the amount of 5.7 × 10^−4^% dry weight) [19], we hypothesized that tricin would also be present in OFG and SFG and it could exert beneficial effects on colorectal cancer treatment. Previous studies demonstrated the inhibitory activities of tricin on self-renewal capacity of colon cancer cells [20] and invasion of C6 glioma cells [21] in vitro, as well as the anti-inflammatory activity in human immune cells [22]. Nevertheless, the potential inhibitory activities of tricin in a metastatic colon tumor animal model have not yet been reported. In the present study, attempts have been made to investigate the inhibitory activities of tricin in human and mouse colon cancer cells as well as in syngeneic orthotopic colon tumor-bearing mouse model. The systemic efficacy of orally administered tricin on tumor metastasis and microenvironment have also been elaborated. 

## 2. Results 

### 2.1. Quantification of Tricin in Medicinal Grains

The tricin content in three medicinal grains was quantified using UPLC. Results showed that the content of tricin in HFG, OFG and SFG were 17.9 ± 1.3, 23.6 ± 2.2, and 21.5 ± 3.8 µg/g, respectively. 

### 2.2. Tricin Reduced Cell Viability and Migration of Human and Mouse Colon Cancer Cells

Cell viability of colon cancer cells after 24 or 48 h tricin treatment was assessed by an MTT assay. Tricin reduced the cell viability of both human and mouse colon cancer cells after 48 h incubation, with IC_50_ values at 107.9 µM and 34 µM in HT-29 and Colon26-Luc cells, respectively (Figure 1D), while tricin (up to 400 µM) did not affect the viability of HT-29 cells and the IC_50_ value for Colon26-Luc cells was greater than 500 μM after 24 h incubation. Tricin was shown to inhibit colon cancer cell motility in scratch wound healing assay. As shown in Figure 1E, the closed wound areas in tricin-treated groups (25 or 50 μM) were fewer than that in untreated group after 24 h in HT-29 cells or 18 h in Colon26-Luc cells. The migration phenotype of Colon26-Luc cells was more obvious than that of HT-29 cells. Nevertheless, the closed wound area (as a percentage of control) was shown to be significantly lower in cells treated with 25 or 50 μM tricin when compared to control (*p* < 0.05, Figure 1F). These data suggested that tricin could significantly decrease the cell motility in Colon26-Luc cells in a concentration-dependent manner. 

### 2.3. Tricin Affected Akt, Erk1/2 and NF-κB signaling Pathways in HT-29 Cells 

**Figure 2:**
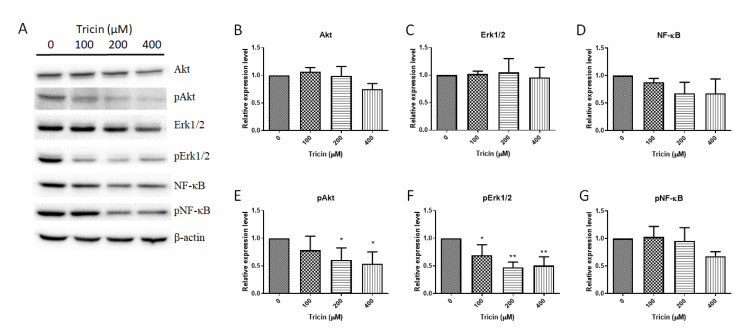
Effects of tricin on protein expressions in HT29 cells. Proteins were extracted from cells incubated with tricin for 24 h and subjected to western blot analysis. (**A**) Representative blots (four protein samples from each group) show the effects of tricin on the expressions of (**B**–**D**) total Akt, Erk1/2, NF-κB and (**E**–**G**) their phosphorylated forms. Original blots are provided in the Supplementary Information. Histograms show the quantitative target proteins expression results normalized with the corresponding β-actin expressions and expressed as relative expression levels. Data were expressed as the mean + S.D. (n = 4). Differences among treatment groups were determined by one-way ANOVA followed by Tukey’s multiple comparisons test, * *p* < 0.05, **p < 0.01 as compared with control group.

As tricin was shown to alter the cell motility of HT-29 cells, its effects on proliferation- and metastasis-related protein expressions were further explored. HT-29 cells were treated with tricin at 100, 200 and 400 μM for 24 h, at which time the viability of HT-29 cells was not affected as mentioned above. As shown in Figure 2, tricin significantly reduced the expressions of phosphorylated protein kinase B (or Akt), phosphorylated extracellular-signal regulated kinase (ERK1/2), nuclear factor-kappaB (NF-κB) and phosphorylated NF-κB in dose-dependent manner (Figure 2A,D–G), while the Akt and Erk1/2 expressions were less sensitive to the tricin treatment (Figure 2B,C).

### 2.4. Tricin Suppressed Mouse Orthotopic Colon Tumor Growth

On the other hand, since Colon26-Luc cells were shown to be more sensitive to tricin treatment when compared to HT-29 cells in the migration assay, and previous studies also reported the immunomodulatory activities of tricin in vitro [22] and in *Apc^Min^* mice [11], syngeneic Colon26-Luc tumor-bearing Balb/c mice model was therefore adopted to reveal the systemic effects as well as the tumor-related immunomodulatory activity of tricin. In particular, the potential inhibitory effect of tricin on metastasis was evaluated in mice, which were orthotopically injected with Colon26-Luc cells and were under real-time monitoring of tumor growth by IVIS during treatment. Tricin was orally gavaged daily at 19 or 37.5 mg/kg, the latter dosage was previously reported to be effective in controlling carcinogenesis in *Apc^Min^* mice [11].

**Figure 3:**
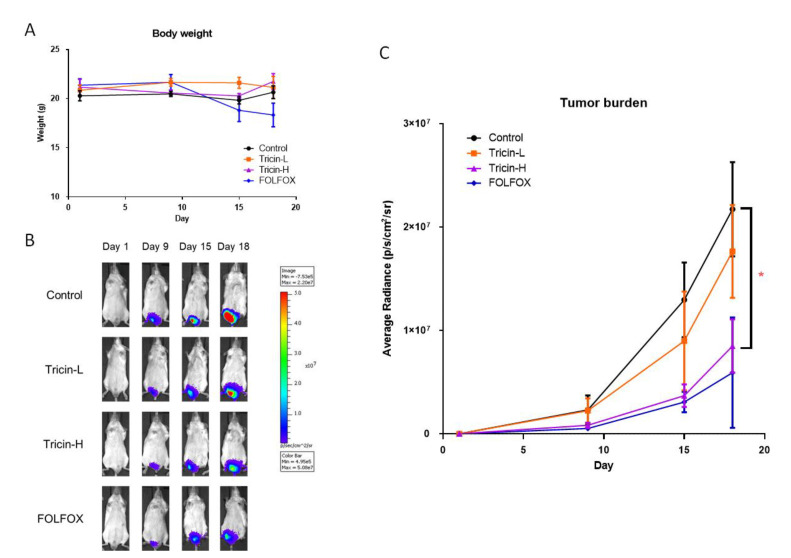
Effects of tricin and FOLFOX on tumor burden in orthotopic colon tumor-bearing mice. Mice were treated with Tricin-L (19 mg/kg), Tricin-H (37.5 mg/kg) or FOLFOX (combination please refer to Methods section) for 18 days. (**A**) Body weights of mice were recorded during the treatment. (**B**) Representative images of mice during treatment at each time point of IVIS scan. (**C**) Bioluminescence measurements according to the average radiance. Data were expressed as mean ± S.E.M., n = 6–13. Differences among treatment groups were determined by one-way ANOVA followed by Tukey’s multiple comparisons test, * *p* < 0.05 as compared with control group.

Results showed that tricin treatments at these two tested dosages did not affect the final body weights of mice (Figure 3A), while the chemotherapeutic treatment FOLFOX slightly decreased the body weight. Tumor growth in the rectum was monitored using IVIS imaging as the colon tumor cells were tagged with luciferase. The tumor burden in terms of bioluminescent signal emission (expressed as average radiance) increased from day 9 onwards in all groups (Figure 3B,C). Treatment with Tricin-H or FOLFOX resulted in obvious inhibitory effects on tumor growth in rectum as lower average radiance was observed in these groups. On day 18, significant smaller tumor size in Tricin-H group was shown when compared with untreated control group (*p* < 0.05, Figure 3C). Besides, the positive control FOLFOX treatment also suppressed the tumor growth, although the differences of the final average radiance between FOLFOX and control groups were not statistically significant (*p* = 0.056, Figure 3C).

### 2.5. Tricin Reduced Orthotopic Colon Tumor Metastasis

**Figure 4:**
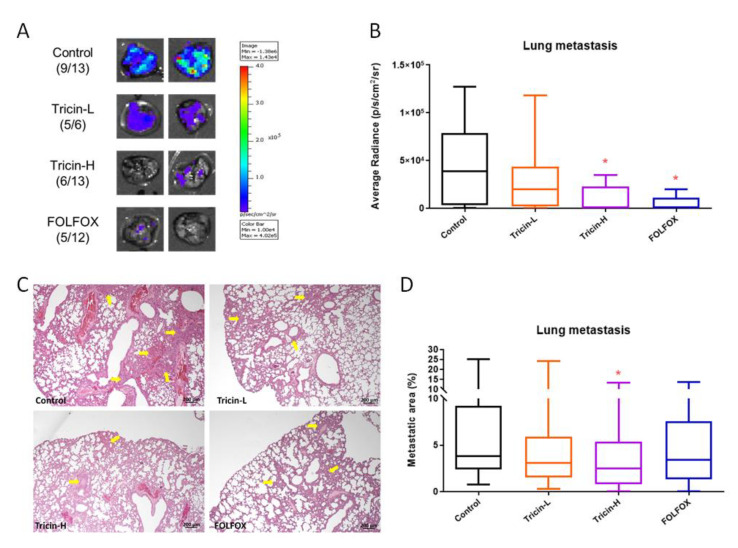
Effects of tricin and FOLFOX on lung metastasis in orthotopic colon tumor-bearing mice. (**A**) Representative images of lungs obtained from different groups at end point of IVIS scan, and the fractional number revealed the frequency of metastasis detected in mice of each group. (**B**) Graph showed the bioluminescence measurements (expressed as average radiance) in lungs. Lungs were also collected and embedded in paraffin. Sections of lungs were stained with H & E and photographed. (**C**) Representative photos showed tumor nodules (yellow arrows) in lung sections. (**D**) Tumor burden were presented as the mean percentage of metastatic area. Mean + S.E.M., n = 7–13 in each group. Differences among treatment groups were determined by one-way ANOVA followed by Tukey’s multiple comparisons test, * *p* < 0.05 as compared with control group.

The metastasis of Colon26-Luc cells in lungs was also examined by bioluminescence imaging. In control group, signal emission from lungs was detected in nine out of thirteen mice (Figure 4A), i.e., 69.2% incidence of metastasis was observed (Figure 4A). Mice received Tricin-H and FOLFOX treatments resulted in lower metastasis incidence, which were 46.2% (6 out of 13 mice) and 41.7% (5 out of 12 mice), respectively (Figure 4A). The average radiance of lungs in Tricin-H and FOLFOX groups was significantly reduced when compared to the untreated control group (*p* < 0.05, Figure 4B). Although the metastasis incidence in lungs from Tricin-L-treated group was high, the overall signal emission was still lower than that of untreated control group (Figure 4B).

In addition, the inhibitory effects of tricin on lung metastasis were further confirmed by histological assessments. Tumor burden in lungs were determined in the paraffin-embedded sections after H&E staining. Figure 4C shows the tumor nodules in lungs of orthotopic colon tumor-bearing mice. The metastasis area was measured and significant decreases of metastatic area in lungs were observed in Tricin-H-treated group (*p* < 0.05, Figure 4D). Overall, FOLFOX treatment was shown to be the most potent in term of inhibition of tumor growth and lung metastasis, while efficacy of Tricin-H treatment could be comparable to that of FOLFOX. Nonetheless, the Tricin-H treatment did not cause body weight loss as FOLFOX treatment did, although the difference between the FOLFOX and control groups was not statistically significant (*p* > 0.05, Figure 3A).

### 2.6. Immune Cell Populations of Syngeneic Colon Tumor-Bearing Mice Altered by Tricin Treatment

**Figure 5:**
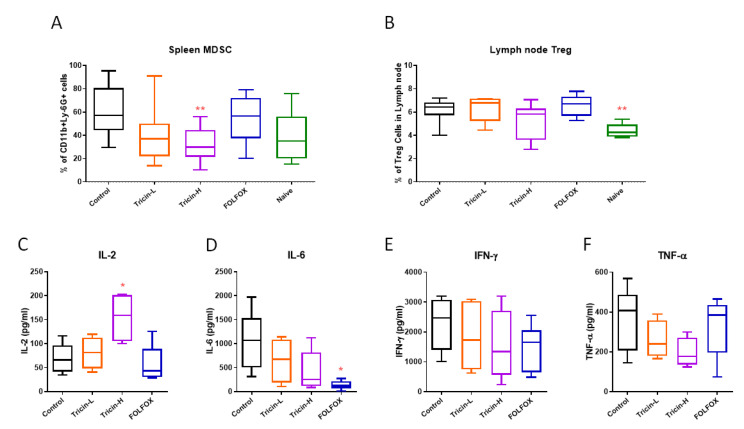
Effects of tricin and FOLFOX on immune cell populations and cytokine productions in orthotopic colon tumor-bearing mice. Spleens and lymph nodes were collected from different groups including naïve group (without tumor cell inoculation). Populations of (**A**) MDSC in spleens and (**B**) Treg in lymph nodes were determined by flow cytometry. Spleen lymphocytes were isolated and then activated with PHA (10 μg/mL). (**C**–**F**) Cell supernatant was collected after 24 h incubation and subjected to ELISA for determination of cytokines (IL-2, IL-6, IFN-γ, TNF-α) productions. Results were expressed as box and whisker plots, n = 7–13. Differences among treatment groups were determined by one-way ANOVA followed by Tukey’s multiple comparisons test, * *p* < 0.05, ** *p* < 0.01 as compared with control group.

Immunocompetent mice bearing syngeneic colon tumors could display the efficacies of tricin or FOLFOX treatments on immunomodulation in mice. Lymphocytes isolated from spleens and lymph nodes from different treatment groups were subjected to flow cytometry to quantify the relative numbers. As shown in Figure 5A, population of myeloid-derived suppressor cells (MDSC) in spleens was higher in tumor bearing control group than that in naïve group (without tumors) (*p* = 0.084). Tricin treatments could reduce the number of MDSC in tumor-bearing mice, with Tricin-H showing significant decrease when compared with control group (*p* < 0.01). Similarly, population of regulatory T (Treg) cells was shown to be significantly higher in tumor bearing mice lymph nodes than that in naïve mice (*p* < 0.05, Figure 5B). Tricin-H treatment also reduced the elevated Treg cell number in tumor-bearing mice, however, the difference between Tricin-H and control groups was not significant (*p* = 0.22). Nevertheless, the ex vivo cytokines productions from mouse spleen lymphocytes were modulated after the mice received different treatments (Figure 5C–F). IL-2 production was higher in Tricin-H group than that in control group (*p* < 0.05). While the pro-inflammatory cytokines IL-6, IFN-γ and TNF-α productions were decreased in tricin-treated or FOLFOX-treated groups, though not statistically significant. 

**Figure 6:**
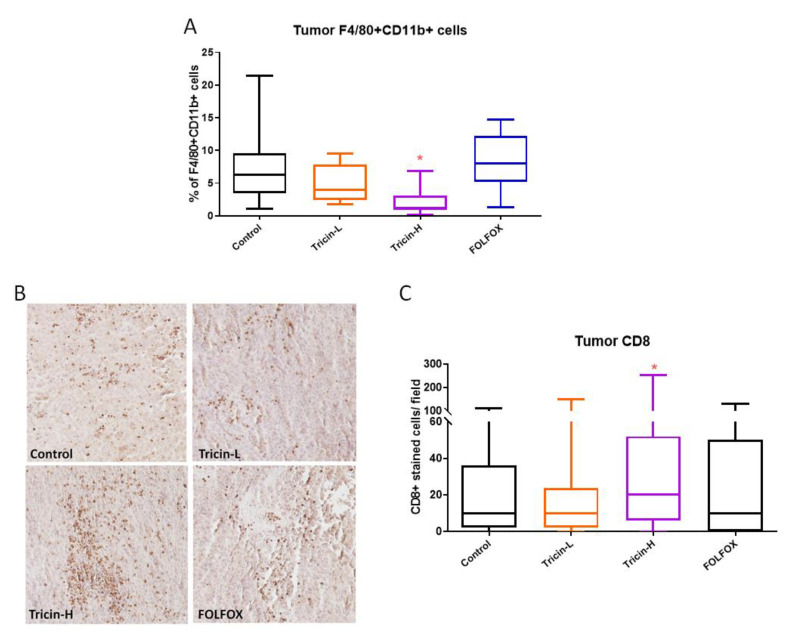
Effects of tricin and FOLFOX on immune cell populations in colon tumors. Tumors were collected from different groups and divided into two halves. Cells isolated from half piece of tumor were stained with anti-F4/80 and anti-CD11b antibodies and analysed by flow cytometry for determination of macrophage population. (**A**) Graph showed the percentage of F4/80^+^ CD11b^+^ stained cells. Another half piece of tumor were embedded in paraffin and the tumor sections were stained with anti-CD8 antibody. (**B**) Representative photos showed CD8^+^ stained cells in dark brown color in the tumor sections. (**C**) Graph showed the number of CD8^+^ stained cells in each field. Results were expressed as box and whisker plots, n = 7–13. Differences among treatment groups were determined by one-way ANOVA followed by Tukey’s multiple comparisons test, * *p* < 0.05 as compared with control group.

On the other hand, the abundancy of macrophages and tumor infiltrated lymphocytes in colon tumors were determined by flow cytometry (with anti-CD11b & anti-F4/80 antibodies) and immunohistochemical staining (with anti-CD8 antibody), respectively. Tricin-H treatment significantly reduced the number of macrophages in tumor (*p* < 0.05, Figure 6A), whereas it significantly increased tumor infiltrated lymphocytes (Figure 6B,C), suggesting the involvement of tricin-modulated immune response against tumor growth and/or metastasis.

## 3. Discussion

Tricin is a flavonoid typically distributed in grasses, grains (rice, wheat, barley, sorghum), bamboo and palms [6,23], with high content in the vegetative tissues (leaves and stem) and bran or husk of cereal/grains [24]. Our chemical analysis showed that tricin was present in 3 tested medicinal grains, HFG, OFG, SFG, which are usually used as Chinese herbal medicines for digestive system disorders. This is the first report of the relatively high amount of tricin found in OFG (23.6 µg/g). This amount was higher than those present in rice bran (10 µg/g) [16] and was comparable to that present in dried leaves of *Festuca* species [10]. In addition, our previous study also showed that OFG water extract (1500 mg/kg) treatments reduced tumor growth and tumor cells metastasis to lungs in orthotopic mouse colon-26 tumor-bearing mice [25]. These data offer some insights into the development of medicinal grains (containing tricin) as health food supplement for colon health. 

Various in vitro regulatory activities of tricin on colon cancer stem cells [20], endothelial cells [26], C2C12 myotubes [27], mesenchymal stem cells [28] and dermal fibroblasts [29] have also been reported. In the present study, tricin was shown to inhibit migration of colon cancer cells HT-29 and Colon26-Luc cells at the non-cytotoxic concentrations. In fact, another human colon cancer cell line HCT-116 has also been tested for tricin’s inhibitory effect on viability and migration (data shown in Supplementary Information). Results showed that the IC_50_ value of tricin in HCT-116 cells was over 600 μM and the significant inhibition on transwell migration could only be achieved by 50 μM or higher of tricin. Thus, HT-29 cells were used for the further experiments. The phosphorylation of key molecules involved in colorectal cancer progression and metastasis, such as Akt [30], Erk1/2 [31] and NF-κB [32], were down-regulated by tricin in HT-29 colon cancer cells, which the results were in line with previous studies. Shalili et al. showed the anti-inflammatory activity of tricin in hPBMCs which was via modulating the p38MAPK and PI3K/Akt pathways [22]. Another study demonstrated that tricin could inhibit proliferation and invasion of C6 glioma cells by downregulating focal-adhesion-kinase (FAK) and thus affecting the downstream Akt pathway [21]. Besides, expression of migration-related proteins, such as integrins, can be evaluated in the future study to illustrate the underlying mechanism of anti-migratory action of tricin.

Recent animal studies demonstrated the broad-spectrum bioactivities of tricin, ranging from anti-obesity effect in high-fatdiet-induced obese mice [33] to protection against UVB-induced wrinkle formation [34]. Nevertheless, the anti-metastatic and/or immunomodulatory effects of tricin in colon cancer model have not yet been reported. Therefore, the present study aimed to verify the systemic efficacy of tricin in treating colorectal cancer using mouse model, of which the promising results might in turn translate to clinical uses. Here, a syngeneic orthotopic colon tumor-bearing mouse model using immune-competent mice was adopted to mimic the clinical pathological condition of metastatic CRC patients. Our results demonstrated apparent anti-tumor and anti-metastatic effects of tricin treatment at 37.5 mg/kg, which was comparable to the effects of chemotherapeutics FOLFOX treatment (Figure 4). The real-time monitoring of the tricin inhibitory effect on orthotopic colon tumor growth was reported here for the first time. This also provided evidence for demonstrating how oral dosage of tricin could gradually control colon tumor progression. Furthermore, in the immune-competent tumor-bearing mice, the ability of modulating tumor immunogenicity of tricin appeared to be stronger than that of FOLFOX since the populations of MDSC in spleens, regulatory T cells in lymph nodes, macrophages and tumor-infiltrating lymphocytes in tumors were altered after Tricin-H but not FOLFOX treatments. It is possible that the immunomodulatory efficacy of tricin was similar to that of its derivative, antartina (tricin 7-*O*-β-D glucopyranoside, isolated from grass *Deschampsia antarctica*) [35]. Intraperitoneal treatment with antartina for three weeks could inhibit colorectal carcinoma growth and liver metastasis in mice. The potent specific cytotoxic T-cell response against colorectal carcinoma and activation of dendritic cells induced by antartina were suggested to be responsible for the anti-tumor effect [35]. Nonetheless, antartina was administered by intraperitoneal injection while tricin was administered by oral gavage in the study. The oral bioavailability of tricin was reported [12] and we showed the efficacy of ingested tricin in colon tumor-bearing mice. Hence, the feasibility to develop tricin as one of the health food ingredients for improving colon health is demonstrated here. Furthermore, based on the immunomodulatory properties of tricin, the potential benefit of combined use of tricin-containing food or medicinal herbs with chemotherapy and/or immunotherapy in colorectal cancer management is worth further investigation.

On the other hand, population changes of MDSC and Treg cells induced by tricin treatment were also revealed in the present study. The tumor microenvironment is composed of tumor cells, fibroblasts, blood vessels, immune cells including T cells, B cells, macrophages, MDSC, dendritic cells, NK cells as well as specific cytokine, chemokines, extracellular matrix milieu, etc. [36]. MDSC have been regarded as promotor of tumor progression through multiple mechanisms in colorectal cancer [37], while Treg was shown to play key role in immunosuppression and cancer progression [38,39]. The multifunctional activities of tricin were now illustrated clearly as the reduction of MDSC and Treg populations and the augmented CD8^+^ T tumor infiltrating lymphocytes population, which is responsible for the anti-tumor effects [40], have been observed in tricin-treated mice. Furthermore, tricin also reduced the number of macrophages in tumors and decreased pro-inflammatory cytokines production by spleen lymphocytes, such changes were consistent with the previous reports on the anti-inflammatory activity of tricin [22,41]. Together with the anti-angiogenic activity reported from other group [26], the important role of tricin on modulating tumor microenvironment was further validated in the immune-competent tumor-bearing mouse model.

In conclusion, basing on our data, tricin appears to be an attractive candidate for future clinical study. Tricin has been shown to be safe for clinical development as a cancer chemopreventive agent [42] and be bioavailable when consumed with the diet in mice [43]. Upon the newly confirmed edible source (i.e., medicinal grains) of tricin, the tricin-containing food can be further developed so that the multifunctional nutraceutical values of tricin can be translated into clinical uses and improve CRC patients’ colon health.

## 4. Materials and Methods

### 4.1. Chemicals and Materials

Flavone tricin (CAS No. 520-32-1, Figure 1A,B, 98% purity based on the 600 MHz ^1^H-NMR spectrum) was purchased from SynInnova Laboratories Inc. (Edmonton, AB, Canada) for the in vitro and in vivo experiments in this study. The chemotherapeutic drugs 5-fluorouracil, folinic acid, and oxaliplatin for animal study were obtained from Sigma-Aldrich (St. Louis, MO, USA). Tricin was dissolved in DMSO and added in culture medium for in vitro or in water (<0.5% *v/v* DMSO) for in vivo experiments.

Human colon cancer cell line HT-29 was purchased from American Type Culture Collection (Manassas, VA, USA). Mouse luciferase-tagged Colon26-Luc cells (JCRB1496) were purchased from Japanese Collection of Research Bioresources Cell Bank (Osaka, Japan). Cell culture media and reagents were all purchased from Thermo Fisher Scientific (Waltham, MA, USA). Male BALB/c mice (6–8 weeks of age) used in this study were provided by Laboratory Animal Services Center of The Chinese University of Hong Kong and were kept under pathogen-free conditions. Raw herbal materials (HFG, OFG, SFG, Figure 1C) were purchased from a renowned supplier in Hong Kong and stored in our temperature- and humidity-controlled storeroom. Raw herbal materials have been morphologically authenticated in accordance with the Chinese Pharmacopoeia 2015. Authenticated voucher specimens (No. 3609, 3610, 3611) were deposited in the museum of the Institute of Chinese Medicine, The Chinese University of Hong Kong.

### 4.2. UPLC Analysis of Tricin in Medicinal Grains 

Stock solution of tricin was prepared in methanol at 1 mg/mL and was stored at −20 °C until use. For preparation of standard curve solution, the stock standard solution was serially diluted into 50, 25, 12.5, 6.25, 3.125, 1.563, 0.7813, 0.391, 0.195, 0.098 µL/mL. For sample preparation, 10 mL methanol was added to 1 g of dry sample powder and was ultrasonicated under 40 °C for 1 h. The supernatant (5 mL) was evaporated to dryness under reduced pressure, followed by 0.5 mL of methanol added to the residue. The mixture was then filtered using a 0.2 µm PTFE filter as the sample solution. The analysis was conducted using a Waters ACQUITY UPLC system (Waters, Milford, MA, USA) The column used was a Waters ACQUITY UPLC BEH C18 1.7 µm, 2.1 × 100 mm, with a Waters ACQUITY UPLC BEH C18 VanGuard 1.7 µm, 2.1 × 5 mm as guard column. The chromatographic separation was conducted at 40 °C under isocratic elution of 0.1% acetic acid in deionized water: acetonitrile (75:25) at a flow rate of 0.35 mL/min. The column was flushed with 100% acetonitrile for 2 min and re-equilibrium for another 2 min after each injection. UV 350 nm was used for the detection of tricin (Figure 1B).

### 4.3. Cell Culture and Viability Assay

Human colon cancer cells HT-29 were grown in McCoy 5A medium supplemented with 10% (*v*/*v*) fetal bovine serum (FBS) and 1% (*v*/*v*) penicillin-streptomycin, while mouse Colon26-Luc cells were grown in RPMI 1640 medium supplemented with 2 mM L-glutamine, 10% (*v*/*v*) FBS and 1% (*v*/*v*) penicillin-streptomycin. Cells were kept in culture flasks in a humidified incubator with 5% CO_2_ at 37 °C. Cells were detached by trypsinization with trypsin-EDTA (0.25%) when they reached 80 % confluence and subcultured every 3–5 days. Cells (5 × 10^3^/well) were seeded into 96-well plates and incubated overnight, then treated with different concentrations of tricin (50–400 μM) for 24 or 48 h. Cell viability was measured using a 3-(4,5-dimethylthiazol-2-yl)-2,5-diphenyltetrazolium bromide (MTT) assay [44].

### 4.4. Scratch Wound Healing and Cell Migration Assays

Cell motility of HT-29 and Colon26-Luc cells were assessed using scratch wound healing assay as previously described [45]. Briefly, 5 × 10^4^ cells were seeded in 24-well plate with full medium and incubated overnight, then starved with medium with 1% (*v*/*v*) FBS for additional 24 h. The cells were scraped using a pipet tip and the scratch wounds in each well were photographed at 0 h. Cell culture medium was changed to fresh full medium with 12.5, 25 or 50 μM of tricin. After 18 h (for Colon26-Luc cells) or 24 h (for HT-29 cells) incubation, medium was discarded and the wounds were photographed again under an IX-71 microscope (Olympus, Tokyo, Japan). The open wound area was measured and analyzed by Tscratch software [46] and the motility of cells was reflected by closed wound area (percentage of control well) after tricin treatment.

### 4.5. Western Blotting of HT-29 Cells

HT-29 cells treated with tricin (100–400 µM) for 24 h were collected for protein. Cells were scraped on ice and then lysed with lysis buffer purchased from Beyotime Institute of Biotechnology (Shanghai, China). Protein concentration was assessed using a BCA kit (Thermo Fisher Scientific, Waltham, MA, USA). Equivalent amount of protein samples, which have been heated at 95 °C, were loaded at 10% SDS-PAGE gels for 2 h at 100 V. Proteins on gel were then transferred to polyvinylidene fluoride (PVDF) membranes (Merck KGaA, Darmstadt, Germany) electrophoretically at 90 V for 1–1.5 h. The membranes were blocked with 5% non-fat milk (*w*/*v*) in Tris-buffered saline Tween 20 (TBST), then primary antibodies were added and the membranes were incubated at 4 °C overnight with gentle shaking. Antibodies against Akt, p-Akt, Erk1/2, p-Erk1/2, NF-κB, pNF-κB (Cell Signaling, Danvers, MA, USA), and β-actin (Sigma-Aldrich, St. Louis, MO, USA) were used. After incubation, membranes were washed with TBST and incubated with horseradish peroxidase (HRP)-conjugated secondary antibodies. Enhanced chemiluminescence solution (GE Healthcare, Chicago, IL, USA) was used to detect the protein bands, which were captured by a molecular imager, ChemiDoc XRS+ (Bio-Rad, Hercules, CA, USA). Signals were quantified by Image J software (NIH, MD, USA) and normalized by β-actin. Quantitative data were represented as relative expression level (fold changes of untreated control). All western blotting assays were independently performed for at least three times.

### 4.6. Syngeneic Orthotopic Colon Tumor-Bearing Mouse Model

The effects of tricin were evaluated in syngeneic orthotopic colon tumor-bearing mouse model, in which the growth of tumor and metastasis of colon cancer cells were monitored using an In Vitro Imaging System (IVIS). The luciferase-tagged mouse cancer cells Colon26-Luc (1 × 10^6^ cells per 40 μL PBS) were injected into the posterior wall of the rectum of BALB/c mouse under anesthesia. Three days after cell inoculation, live animal whole-body imaging to monitor luciferase-expressing cells in mice was performed to ensure the successful inoculation using IVIS 200 (Xenogen, Alameda, CA, USA). Images were acquired for 1 to 180 s and the photon emission transmitted from each mouse were quantified using Xenogen Living Image (Igor Pro version 3.2 software), and the data were processed to produce graphs according to the average radiance (photons/s/cm^2^/sr) [47]. The tumor-bearing mice were randomized into different groups and received tricin at 19 or 37.5 mg/kg (Tricin-L and Tricin-H, respectively) by oral gavage daily for 18 days (n = 6–13). A group of mice received FOLFOX (7.5 mg/kg fluorouracil and 2.5 mg/kg folinic acid daily for 18 days plus 2.5 mg/kg oxaliplatin once a week) injection intraperitoneally was served as positive control group. The IVIS imaging was performed on Days 1, 9, 15, 18 during treatments so as to monitor the growth of orthotopic tumors. At the end of experiment, mice were undergone IVIS imaging and then sacrificed under anesthesia. Then the lungs were excised for IVIS imaging because the bioluminescence signal of the metastasized Colon26-Luc cells in lungs could not be detected in situ. Meanwhile the spleens, lymph nodes and half of the tumors were collected for isolation of immune cells for further analyses. The lungs were also subjected to histological examination for metastasis level assessment after haematoxylin & eosin (H & E) staining [48]. Another half of the tumors were subjected to immunohistochemistry staining against CD8 for tumor infiltrating lymphocytes as previously described [49]. The animal experiments were approved by the Animal Experimentation Ethics Committee of The Chinese University of Hong Kong (Ref. No. 18/231/MIS). All methods were performed in accordance with the relevant guidelines and regulations.

### 4.7. Flow Cytometry for Immune Cells Characterization and ELISA for Cytokines Determination

To isolate cells from spleens, lymph nodes and tumors, organs were homogenized by a plunger of a syringe and then passing through a 200-mesh sieve. Isolated cells were blocked twice in PBS with 5% FBS and stained with PE-anti-mouse CD 11b and FITC-anti-mouse Ly-6G (Gr-1) obtained from BD Pharmingen (San Jose, CA, USA) for myeloid-derived suppressor cells (MDSC); or with FITC-anti-mouse CD11b, APC anti-mouse F4/80 obtained from Biolegend (San Diego, CA, USA) for macrophages; or with FITC-anti-mouse CD11b, APC-anti-mouse-CD25 and PE-anti-mouse Foxp3 obtained from eBioscience (Thermo Fisher Scientific) for regulatory T (Treg) cells or IgG isotype for 30 min at 4 °C in the dark. Then cells were washed twice with blocking buffer, fixed with 4% paraformaldehyde and analyzed by flow cytometer (FACSCanto II, Becton Dickinson, Franklin Lakes, NJ, USA). Data from 10,000 cells were collected and analyzed. Lymphocytes isolated from spleens were counted, seeded in 96-well plate at a density of 3 × 10^6^/well, activated with PHA (10 μg/mL) and cell supernatant was collected after 24 h incubation at 37 °C. Enzyme-linked immune sorbent assays (ELISA) kits of mouse IL-2, 6, TNF-α and IFN-γ (BD Biosciences, San Jose, CA, USA) were performed according to the manufacturer’s instructions.

### 4.8. Statistical Analysis

Data of in vitro experiments were presented as means ± standard deviation (S.D.) for at least three independent times. In vivo experiment results were expressed as means ± standard error of the mean (S.E.M.). Quantitative results were analyzed by one-way ANOVA with Dunnett test or Tukey’s *post-hoc* tests. In all comparison, statistical significance was considered when *p* < 0.05. All statistical analysis was assessed by GraphPad Prism 8.0 software package (GraphPad Software Inc., San Diego, CA, USA).

## Supplementary Materials

The following are available online. Supplementary information –HCT116 cells MTT and transwell migration assay results; Supplementary information WB blots.
