# Supplementary material for: A Natural Flavone Tricin from Grains Can Alleviate Tumor Growth and Lung Metastasis in Colorectal Tumor Mice
Source: Molecules. 2020 Aug 15;25(16):3730. doi: 10.3390/molecules25163730 (PMC7463810; doi:10.3390/molecules25163730)
Supplement: Supplementary file 1 [file molecules-25-03730-s001.pdf]

A natural flavone tricin from grains can alleviate tumor growth and lung metastasis in colorectal tumor mice

Grace Gar-Lee Yue[1,2], Si Gao[1,2], Julia Kin-Ming Lee[1,2], Yuk-Yu Chan[3], Eric Chun-Wai Wong[1,2], Tao Zheng[1,2], Xiao-Xiao Li[3], Pang-Chui Shaw[1,2,3,4], Monique S. J. Simmonds[5], Clara Bik-San Lau[1,2,3,*]

[1] Institute of Chinese Medicine; [2] State Key Laboratory of Research on Bioactivities and Clinical Applications of Medicinal Plants (CUHK); [3] Li Dak Sum Yip Yio Chin R&D Centre for Chinese Medicine; [4] School of Life Sciences, The Chinese University of Hong Kong, Shatin, New Territories, Hong Kong. [5] Royal Botanic Gardens, Kew, Richmond, Surrey TW9 3AB, United Kingdom.

A natural flavone tricin from grains can alleviate tumor growth and lung metastasis in colorectal tumor mice

Supplementary information –HCT116 cells MTT and transwell migration assay results

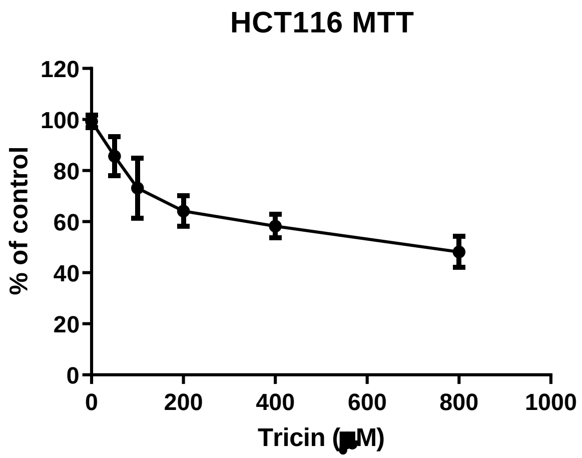

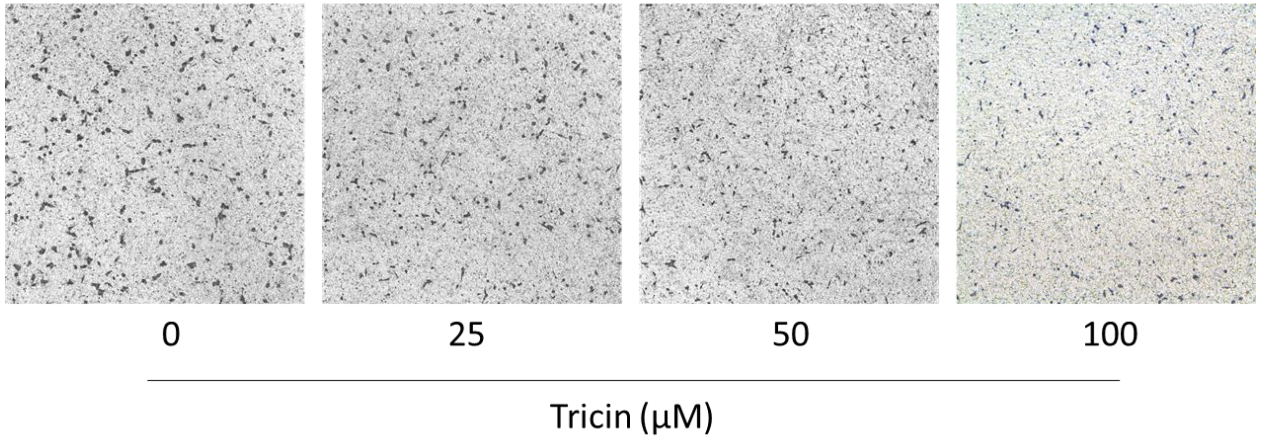

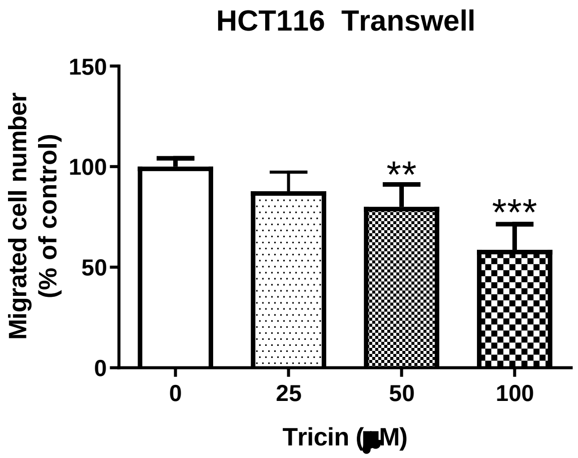

A natural flavone tricin from grains can alleviate tumor growth and lung metastasis in colorectal tumor mice

Supplementary information –WB blots

Figure 2

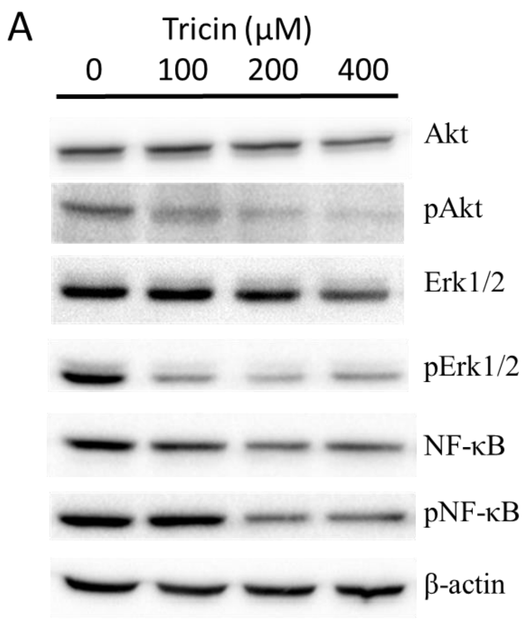

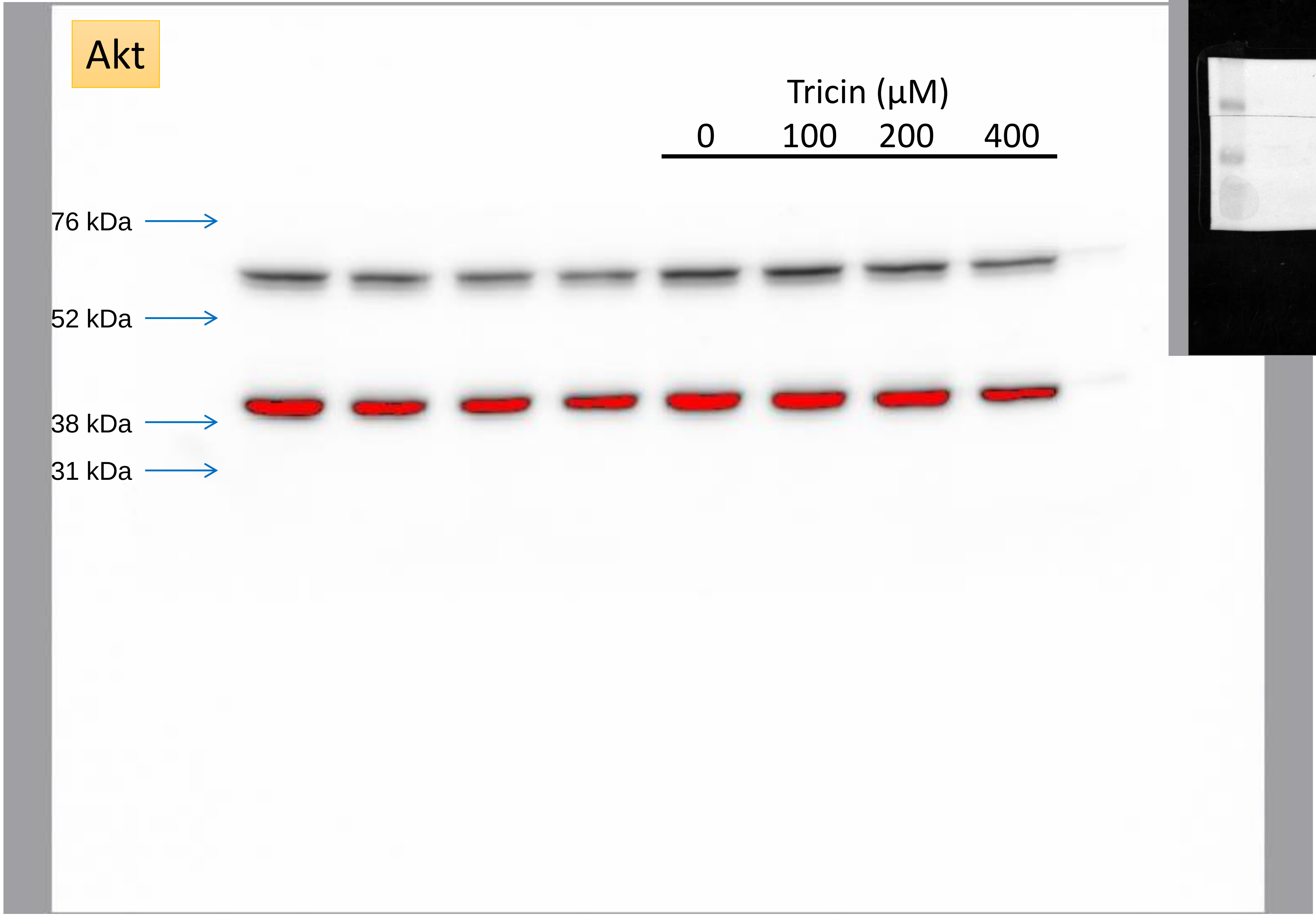
Akt
Tricin (µM)
0
100
200
400
76 kDa
52 kDa
38 kDa
31 kDa

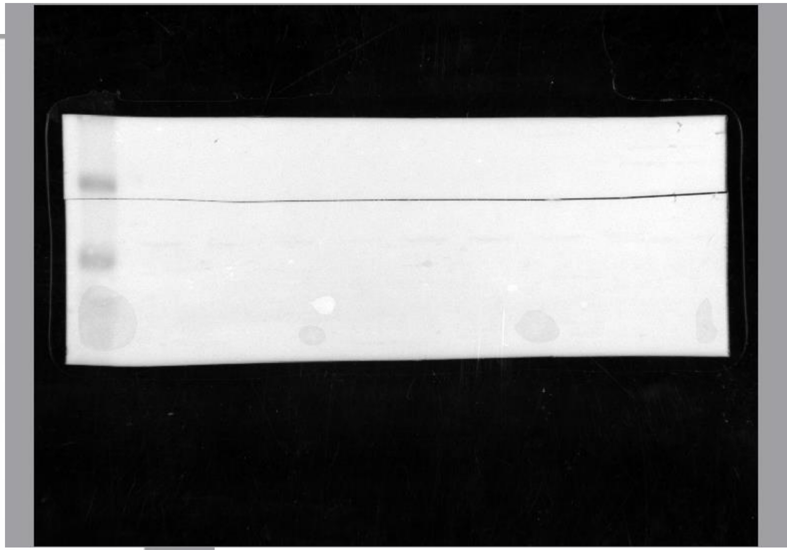

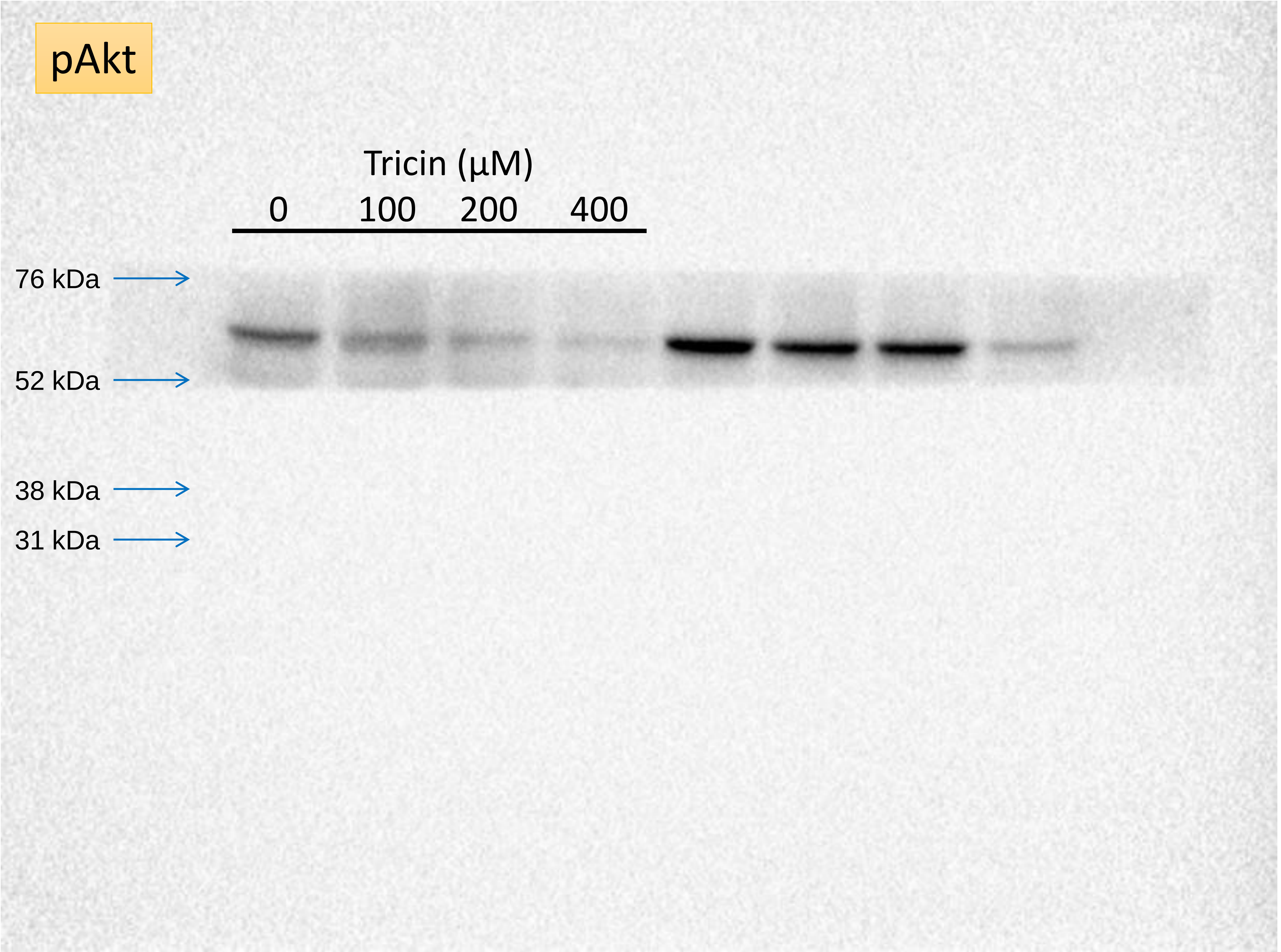
pAkt
Tricin (µM)
0
100
200
400
76 kDa
52 kDa
38 kDa
31 kDa

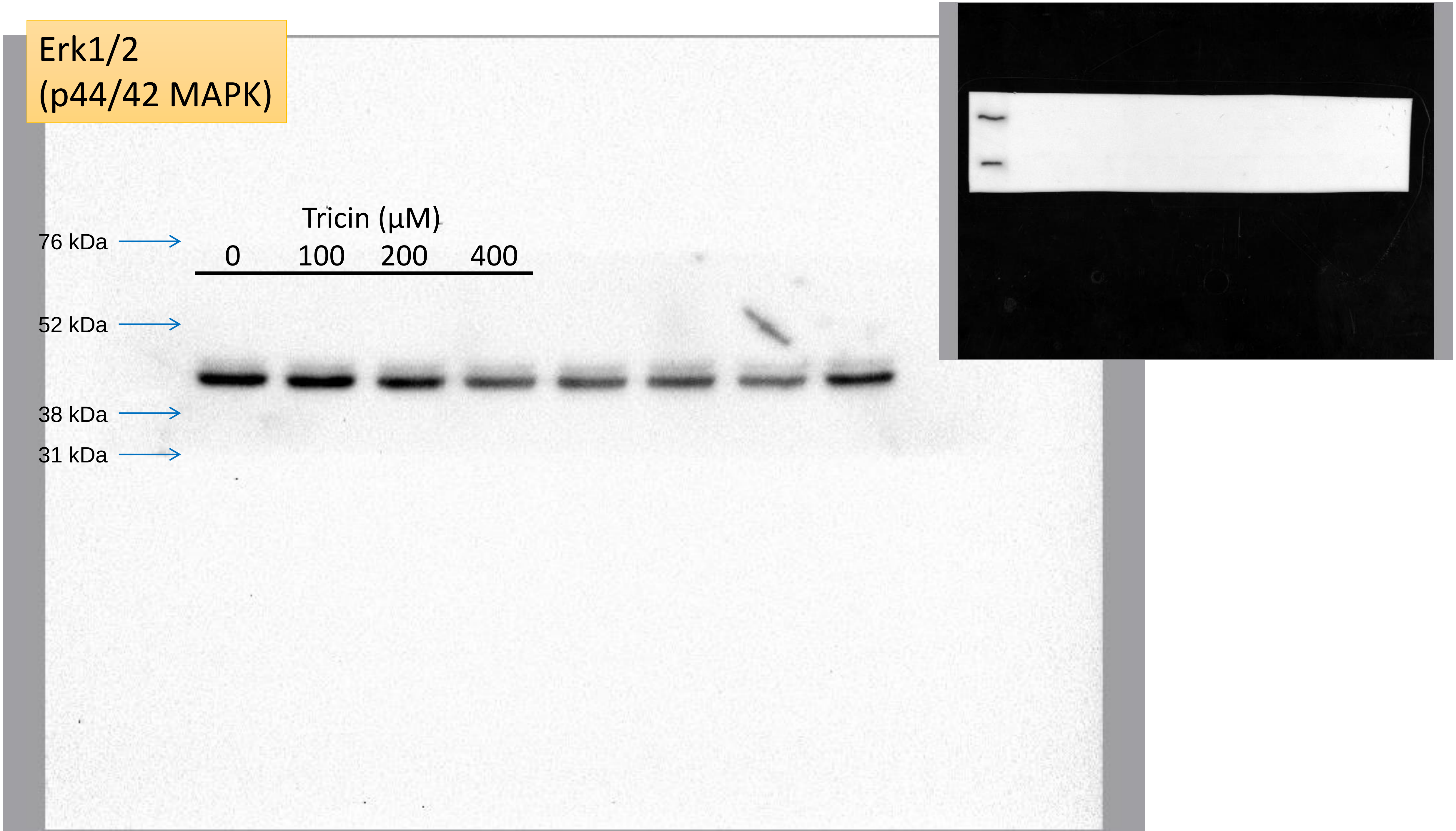
Erk1/2
(p44/42 MAPK)
Tricin (µM)
0
100
200
400
76 kDa
52 kDa
38 kDa
31 kDa

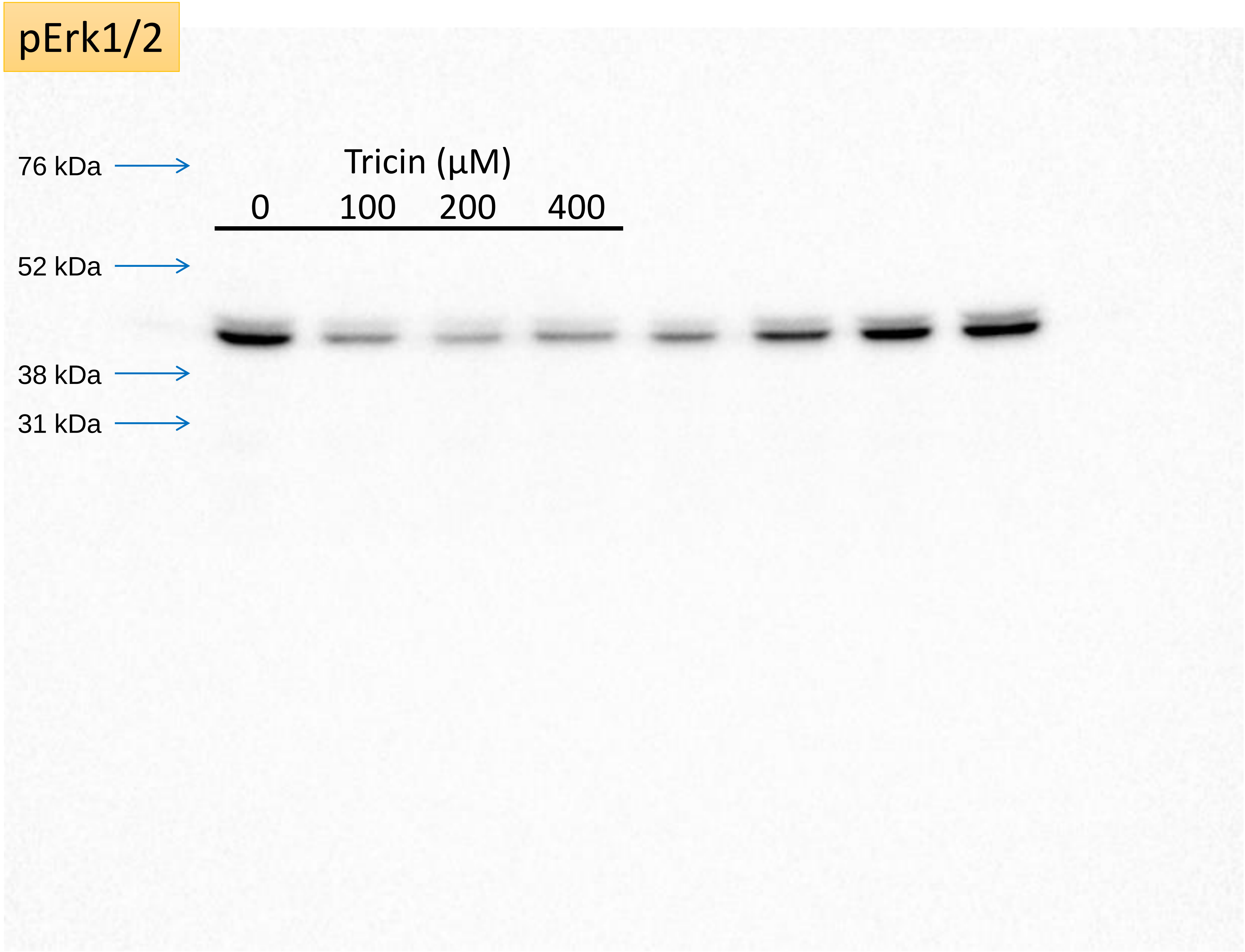
pErk1/2
76 kDa
52 kDa
38 kDa
31 kDa
Tricin (μM)
0
100
200
400

NFκB

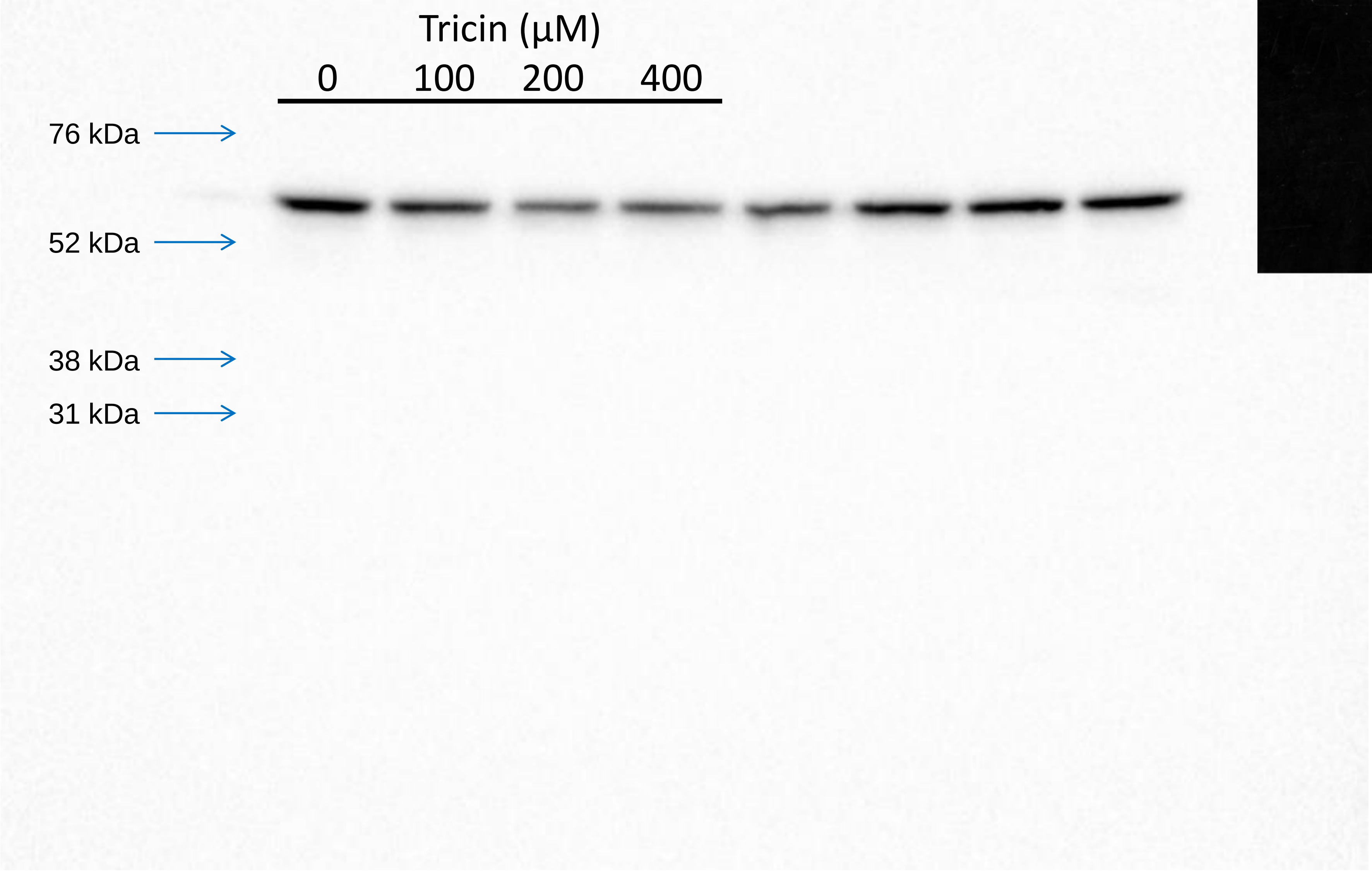

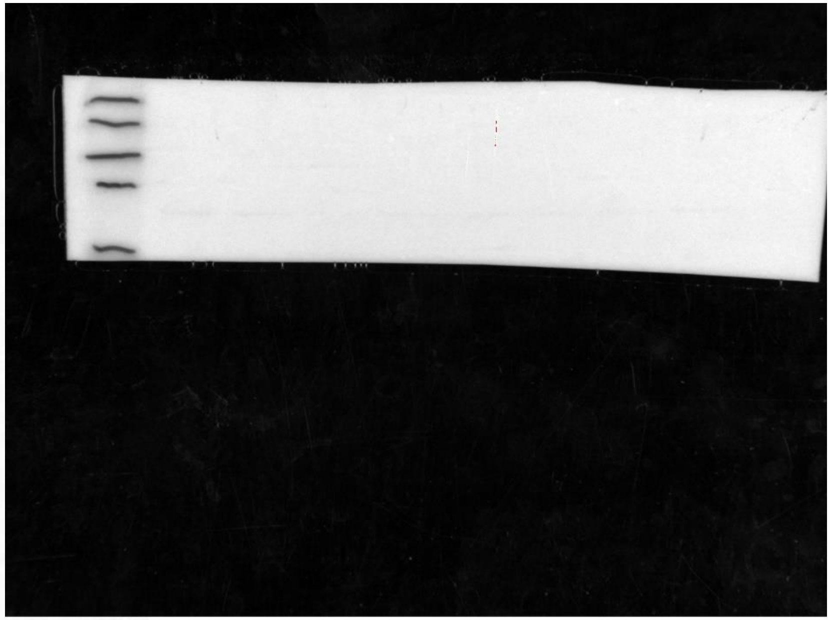

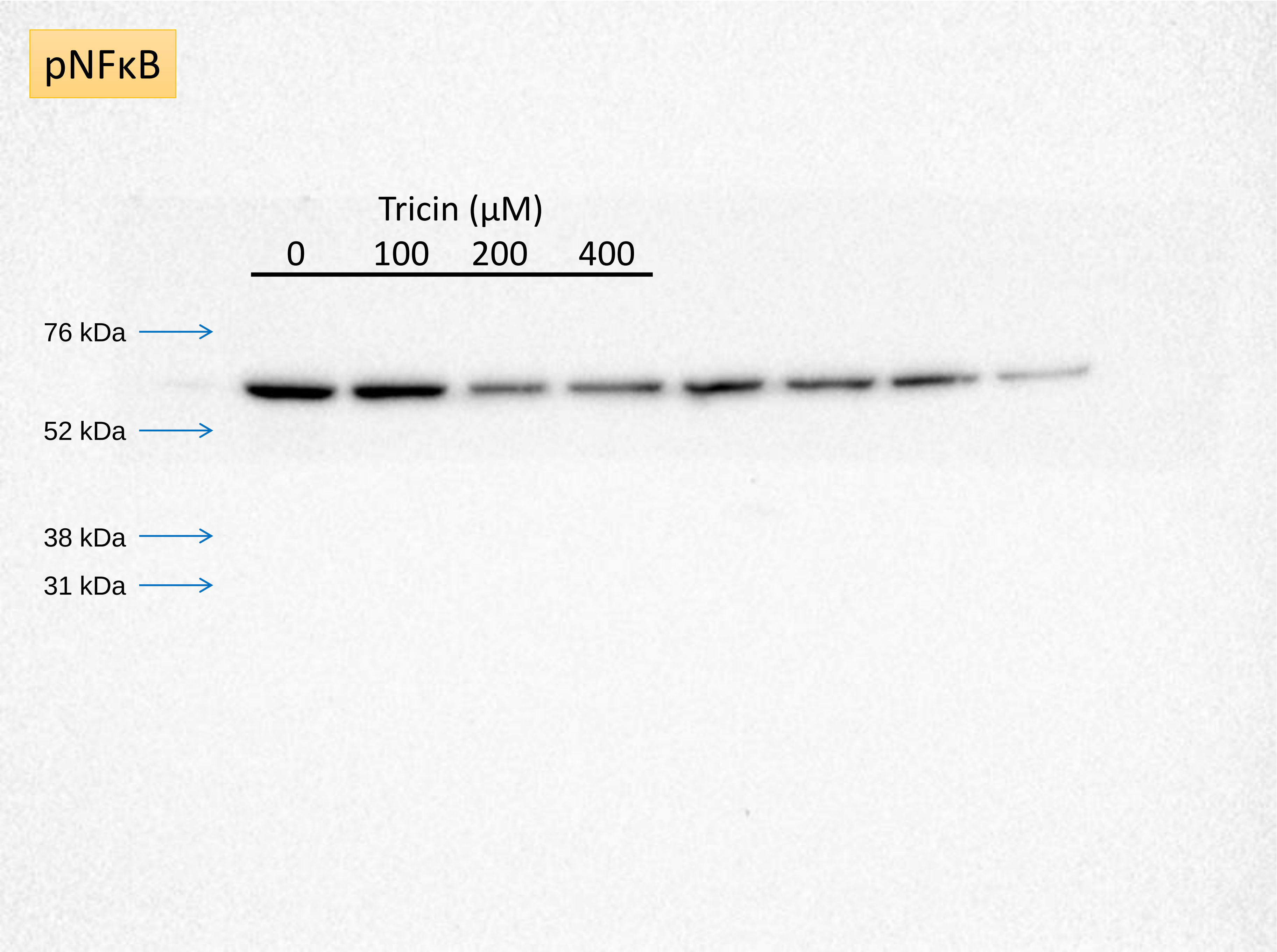
pNFκB
Tricin (μM)
0
100
200
400
76 kDa
52 kDa
38 kDa
31 kDa
